# Pediatric Differentiated Thyroid Cancer: Our Experience

**DOI:** 10.7759/cureus.4693

**Published:** 2019-05-17

**Authors:** Wafaa Abd Elhameed Elsayed, Mahmood A Hamed, Rasha A Ali, Rafaat A Bakheet

**Affiliations:** 1 Nuclear Medicine, Sohag University Hospital, Sohag, EGY; 2 Otorhinolaryngology, Sohag University Hospital, Sohag, EGY; 3 Epidemiology and Public Health, Sohag University Hospital, Sohag, EGY; 4 Oncology, Sohag University Hospital, Sohag, EGY

**Keywords:** differentiated thyroid cancer, pediatric, treatment, radiation, outcome

## Abstract

Objectives: To report our experience in the management of thyroid cancer in children and adolescents in a tertiary referral hospital and regional cancer institute as compared to previously published data.

Methods: A retrospective study was conducted for patients diagnosed with differentiated thyroid cancer (DTC) who received treatment during the period from January 2014 to August 2018. Medical reports from our hospital database were extracted and information of those under 18 years old were discussed regarding their demographics, treatment received, and follow-up outcomes.

Results: Out of 300 patients with DTC diagnosed in the period of study, 12 were 18 years old or less (4%). Female to male ratio was 5:1. Their ages ranged from nine to 18 years old (average: 13.1 years). One patient had a positive family history for DTC, and one patient had lung metastasis. Total thyroidectomy and postoperative ^131^I were performed for all patients. The median follow-up period was 1.75 years (range: six months to four years). Eleven patients have shown complete remission after treatment (91.6%), and one case has had persistent disease.

Conclusions: Pediatric thyroid cancer is not uncommon. Despite its aggressiveness in this age group, outcomes are more favorable than in adults. We report our experience in the diagnosis and management of pediatric DTC in our community with satisfactory outcomes and comparable results to literature reports. Future studies are needed to evaluate the long-term complications of radioiodine therapy.

## Introduction

Differentiated thyroid cancer (DTC) in children and adolescents is much less frequently encountered in practice as compared to adults [1-2]. Although most cases tend to be more aggressive, they have a generally better prognosis and response to therapy than those presented in adulthood, even when associated with extrathyroidal involvement [3-5]. Female gender carries a higher risk with a higher incidence discovered among females during the adolescent age group (10 - 18 years) [6-7]. The papillary type represents the highest ratio (about 90%) [7], followed by the follicular type (10% or less) [8-9]. In this work, we describe our experience with pediatric cases of DTC managed at our university-affiliated hospital and regional cancer institute over a four-year duration, discuss their epidemiologic aspects, and methods of diagnosis, treatment, and follow-up outcomes.

## Materials and methods

This study complies with the ethical, regional and institutional guidelines for medical research, as well as with the Declaration of Helsinki. A retrospective study was performed for patients diagnosed with differentiated thyroid cancer who received treatment in our tertiary medical institute and cancer institute during the period from January 2014 to August 2018. Medical charts from the hospital database for those patients were reviewed and the following data were retrieved:

· Patient demographics (age at the time of diagnosis, gender, clinical presentation, and family history of thyroid cancer),

· Histopathological type of thyroid cancer,

· Clinical staging,

· Treatment received (surgery, radioactive iodine),

· Follow-up (every six months by thyroglobulin, thyroid-stimulating hormone (TSH), thyroid ultrasound, and when indicated, diagnostic whole body scan).

All of our patients received radioactive iodine ^131^I. In order to facilitate ^131^I uptake by residual iodine-avid cancer, the TSH level should be above 30 mIU/L. This was achieved in all children by withdrawing levothyroxine (LT4) for 21 days and instituting a low iodine diet. Adjustment of ^131^I activity was calculated according to weight and then given a fraction (e.g., the child’s weight in kilograms/70 kg) based on the typical adult activity used to treat residual disease or disease extent [10]. Seven days following ^131^I therapy, we performed a whole-body scan to take advantage of the increased sensitivity associated with administration of the larger activity of ^131^I used for treatment [11]. Short-term side effects were reported in two cases as neck pain/stomatitis which responded to medical treatment The use of sour candy or lemon juice, starting after ^131^I dosing, with vigorous hydration for three to five days was helpful to decrease sialadenitis [12].

Statistical analysis

Qualitative data were summarized as numbers and percentages, whereas quantitative data were summarized as range, median, and averages.

## Results

From January 2014 to August 2018, 300 patients were diagnosed as differentiated thyroid carcinoma. Of those patients, 12 were children and adolescents up to the age of 18 years old (4%). Of this pediatric age group, 10 were females (83.3%) and two were males (16.7%). Their ages ranged from nine to 18 years old (median = 13 years, average = 13.1 years). Only one patient was below 10 years. All cases were chiefly presented by painless neck swelling of variable duration.

Only one female had a positive family history for thyroid cancer (first-degree diagnosed as papillary thyroid carcinoma). In regard to the pathological types, the classic variant of papillary carcinoma was found in 10 patients and a follicular variant in two patients. Concerning the risk stratification, nine patients were low-grade (75%), two were intermediate-grade (16.6%), and one was high-grade (8.3%). Concerning treatment, a total thyroidectomy was performed in all patients with nodal dissection performed for three patients (25%). Radioactive iodine ^131^I was given to all patients after surgery as a single dose (range: 30 to 80 ml curie for each patient) according to the age and body weight, except for the patient with lung metastasis for another dose. Median follow-up period was 1.75 years (range: six months to four years).

Eleven patients have shown complete remission after treatment (91.6%), and one case has had persistent disease (this patient had lung metastasis from the start). 

A summary of the patients’ data is shown in Table 1.

**Table 1:** Pediatric Thyroid Cancer Patients Over a Four-year Duration at Our University Hospital CR: complete remission; F: female; LN: lymph node; M: male; RAI: radioactive iodine; -ve: negative family history; +ve: positive family history

ID	Age (years)	Sex	Pathology	Surgery	RAI	Follow-up period	Outcome	Family history	Risk stratification
1	10	F	papillary	total thyroidectomy + LN dissection	Yes	4 years	CR	+ve	Intermediate
2	9	F	follicular variant of papillary	total thyroidectomy + LN dissection	Yes	6 months	CR	-ve	Intermediate
3	17	M	papillary	total thyroidectomy	Yes	2 years	CR	-ve	Low
4	11	F	papillary	total thyroidectomy	Yes	3 years	CR	-ve	Low
5	15	F	follicular variant of papillary	total thyroidectomy	Yes	1.5 years	CR	-ve	Low
6	12	F	papillary	total thyroidectomy	Yes	3 years	CR	-ve	Low
7	14	F	papillary	total thyroidectomy	Yes	1 year	CR	-ve	Low
8	10.5	F	papillary	total thyroidectomy	Yes	1 year	CR	-ve	Low
9	14	M	papillary	total thyroidectomy + LN dissection	Yes	6 months	Persistent lung metastasis	-ve	High
10	18	F	papillary	total thyroidectomy	Yes	2 years	CR	-ve	Low
11	16	F	papillary	total thyroidectomy	Yes	1 year	CR	-ve	Low
12	11	F	papillary	total thyroidectomy	Yes	2.5 years	CR	-ve	Low

## Discussion

The annual incidence of DTC among adults is much higher than that seen in the children and adolescent age groups. However, there is recent evidence supporting that this incidence is increasing [10]. It increases gradually with age, reaching its peak between 20 - 30 years. Female gender carries a higher incidence, particularly in the postpubertal period, whereas no sex difference exists in the prepubertal period [1-2, 6, 13]. In Egypt, thyroid cancer represents about 1.5% of all cancers in both sexes and 3.6% of female malignancies and constitutes about 30% of endocrine malignancies with overall female to male ratio less than three [14]. This study complies with previous reports concerning this issue. Among 300 patients with DTC, we had 12 patients aged 18 years or less (4%) with only one patient below the age of 10 years and a female to male ratio of 5:1. In contrast to previously published reports [9, 13, 15], our series showed most cases presented early with painless neck swelling and their staging was low (9/12 cases). Nodal involvement was observed in three cases, whereas one case had lung metastasis. The diagnosis was established by neck imaging and fine needle aspiration cytology (FNAC) which matches with updated recommendations [10]. Owing to variations in the clinical picture, pathology, and long-term outcomes, treatment strategy in children differs from adult thyroid cancer [13]. Our strategy for management of thyroid cancer is in agreement to a great extent with the American Thyroid Association (ATA) guidelines for pediatric thyroid cancer published in 2015 [10]. Total thyroidectomy was performed for all patients, even in the early stages, without nodal involvement or extrathyroidal spread. Evidence supports that total thyroidectomy is appreciated over conservative lobectomy in childhood DTC as it improves locoregional control and allows eradication of multifocal disease, which is not uncommon. In addition, the likelihood of recurrence is much lower [16-17].

Thyroid surgery is best performed by an experienced thyroid surgeon to decrease the risks of permanent hypoparathyroidism, and neural monitoring of the recurrent laryngeal nerve should be used to reduce the risk of permanent vocal cord paralysis [17-18]. In our series, such complications were not encountered.

For ^131^I therapy of pediatric papillary thyroid carcinoma (PTC), many parameters are even more important than the administered activity for the radiation absorbed dose to tumor lesions, such as ^131^I avidity of tumor tissue, residence times, the effective ^131^I half-life, the size, and the shape of the tumor [19-20].

For pediatric patients receiving RAI, two approaches are known. The first approach, developed by Benua et al. [21-22], aimed to determine the activity that is as high as safely administrable (AHASA), thus targeting the safety limits of the absorbed dose to the blood to 2 Gy and the whole body activity in RAI in adults to 80 mCi or 3 GBq ^131^I at 48 hours after the administration because this will help to avoid severe damage to the hematopoietic system and lung complications, such as pneumonitis or pulmonary fibrosis [21-22].

In the second approach, Maxon et al. [23] were targeting efficacy. To our knowledge, no prospective studies in pediatric PTC patients focusing on long-term recurrence and survival have been conducted. The precise identification of which patients should receive ^131^I therapy will remain a subject of debate and controversy. Pediatric DTC behaves differently than adult DTC and, for this reason, should be treated differently. Long-term follow-up studies are crucial to increase the knowledge of the clinical behavior of pediatric DTC, risk factors, recurrence, and late effects of the administered treatment.

## Conclusions

Herein, we reported our experience in the diagnosis and management of pediatric DTC in our community with favorable outcomes and comparable results to literature reports. Out of 300 patients, 12 were children and adolescents (4%); 11 patients had a complete remission and only one patient of the 12 still has lung metastasis. Our management plan and follow-up were compatible with ATA guidelines. However, larger studies and extended follow-up periods are strongly needed to evaluate long-term sequelae and delayed complications of the treatment received, especially with radioactive iodine.
